# Urogenital infections select for reduced ability to grow in a low-iron environment and reduced blood survival for Staphylococcus aureus

**DOI:** 10.1099/mic.0.001691

**Published:** 2026-06-08

**Authors:** Kate P. Kearney, Elizabeth V. K. Ledger, Mario Recker, Ruth C. Massey

**Affiliations:** 1 School of Microbiology, UCC, Cork, Ireland; 2 APC Microbiome Ireland, UCC, Cork, Ireland; 3 Centre for Ecology and Conservation, Penryn Campus, University of Exeter, Penryn, UK; 4 Institute for Tropical Medicine, University of Tübingen, Tübingen, Germany; 5 School of Cellular and Molecular Medicine, University of Bristol, Bristol, UK

**Keywords:** bacteraemia, iron dependency, *Staphylococcus aureus*, urogenital

## Abstract

Immunity through nutrient sequestration is one of the means by which a host can protect itself from infection. As a consequence, many micro-organisms have evolved strategies to overcome this, including the opportunistic pathogen *Staphylococcus aureus*. In this study, we focussed on a population of SAB isolates with the aim of better understanding their ability to grow in a low-iron environment and observed a significant association between a weakened ability to grow in a low-iron environment and whether the entry point of the bacteria into the bloodstream was urogenital in origin. Follow-up experiments demonstrated that urine at 10% provided sufficient iron for both a defined iron uptake mutant (*sbnE::*Tn) and our iron-dependent clinical isolates to grow, in the absence of other sources of iron. This potentially explains their association with this environment where, although at relatively low levels, the iron there is such that those with poor iron utilization ability can survive. This does not however explain how these isolates went on to successfully cause bacteraemia, given how poorly available iron is in the bloodstream. To determine whether these iron-dependent isolates had some alternative means of surviving in blood, we exposed them to fresh human blood and found that although they had previously all successfully established an infection in the bloodstream, the iron-dependent isolates did not survive as well as the non-dependent isolates. This work highlights the complexity and apparent contradictory nature of the factors that contribute to the development of this important disease.

## Introduction

*Staphylococcus aureus* is a major human pathogen that causes infections that vary in both body site and severity [1,2]. One of the most severe types of infections it causes is bacteraemia, with a mortality rate of between 20 and 30% [3]. Of significant concern here is that in spite of all recent advances in modern medicine, the incidence of *S. aureus* bacteraemia (SAB) is increasing in many countries, for reasons we do not understand [4]. The onset of SAB is multifactorial, as are the features that affect patient outcome [1]. Often the site of entry of the bacteria into the bloodstream is known, and depending on where this is, it can be readily addressed and lead to the resolution of the infection, i.e. the removal of an infected catheter [1,5]. While some bacterial and host factors that contribute to poor patient outcomes are known, for example, high levels of cytolytic toxin production by bacteria and advanced patient age, these do not fully explain why some infections resolve and others do not [6,7]. This highlights major gaps in our understanding of *S. aureus* pathogenesis and the determinants of patient outcome.

Nutritional immunity is a means by which the host can sequester important nutrients away from invading pathogens, thus limiting their ability to replicate and cause disease [8,9]. Many pathogens, including *S. aureus*, have evolved a range of strategies to get around this. Iron, for example, is a critical micronutrient for bacterial growth as it is a vital cofactor for many proteins including cytochromes and iron-sulphur cluster proteins but is rarely found in a free form in the human body and is instead sequestered within molecules such as haem and transferrin [9,11]. *S. aureus* can access this sequestered iron using multiple strategies, including the release of siderophores that capture this iron and bring it back to the bacterial cell for use, as well as the expression of the Isd uptake machinery within its cell envelope [8,10]. Given the importance of this activity to the ability of *S. aureus* to establish an infection in the bloodstream, we sought to examine whether there was much variation in their ability to do this and whether any identified variability was associated with patient outcome.

To address this, we examined the ability of 134 bacteraemia isolates, all belonging to a single clonal complex (CC22), to grow under iron-limited conditions [12]. The CC22 collection represents a globally important clonal type which includes clinical *S. aureus* isolates, isolated from patients with bloodstream infections across the UK and Ireland [12]. We found that there was a wide range of abilities of the bacteria to grow in a low-iron environment within this collection. However, despite the genomes being available for these isolates, we were unable to identify any specific loci as contributing to this variability. While we detected no association between the ability to grow in a low-iron environment and patient mortality, isolates that entered the bloodstream via a urogenital route were most likely to be iron dependent, a feature we believe is due to them losing their energetically costly iron-scavenging activity due to the level of iron in urine being sufficient to support their growth without this [13]. These iron-dependent isolates were however also significantly impaired in their ability to survive in an *ex vivo* blood model, which was unexpected given that they were isolated from the bloodstream of bacteraemic patients. Together, these findings indicate that the location from which *S. aureus* enters the bloodstream can significantly impact the behaviour and pathogenic capabilities of the bacteria within the bloodstream, further highlighting the complexity of the factors contributing to the success of this important pathogen.

## Methods

### Bacterial strains and growth conditions

A list of *S. aureus* strains used in this study can be found in Table 1. The clinical isolates used are described in Recker *et al*. [12]. Strains were grown on tryptic soy agar (TSA) or in tryptic soy broth (TSB) with shaking (180 r.p.m.) for 17 h at 37°C. Nebraska Transposon Mutant Library (NTML) mutants were selected for using erythromycin (10 µg ml^−1^).

**Table 1.** *S. aureus* isolates used in this study

Strain	Description	Reference
*S. aureus* USA300 JE2 WT	Los Angeles County (LAC) strain of the USA300 community-associated methicillin-resistant *S. aureus* (CA-MRSA) lineage cured of plasmids	[14]
JE2 *sbnE*::Tn	JE2 with the bursa aurealis transposon inserted into *sbnE*; Ery^R^	[14]
ASARM68	MRSA, CC22 isolate urogenital in origin	[12]
ASARM79	MRSA, CC22 isolate urogenital in origin	[12]
ASARM95	MRSA, CC22 isolate urogenital in origin	[12]
ASARM100	MRSA, CC22 isolate urogenital in origin	[12]
ASARM116	MRSA, CC22 isolate urogenital in origin	[12]
ASARM118	MRSA, CC22 isolate urogenital in origin	[12]
ASARM120	MRSA, CC22 isolate urogenital in origin	[12]
ASARM191	MRSA, CC22 isolate urogenital in origin	[12]
ASARM209	MRSA, CC22 isolate urogenital in origin	[12]
ASASM12	Methicillin-susceptible *S. aureus* (MSSA), CC22 isolate urogenital in origin	[12]

### Iron limitation growth assay

To prepare an iron-restricted media, RPMI 1640 with 1% casamino acids was treated with Chelex-100 resin (5 g/100 ml) for 1 h at 24°C with shaking (180 r.p.m.). Where appropriate, the following essential ions were re-introduced: ZnCl_2_ (25 µM), CaCl_2_ (100 µM), MgCl_2_ (1 mM) and MnCl_2_ (25 µM) [14]. Where necessary, iron was added in the form of FeCl_3_ (25 µM). For inoculum preparation, colonies were scraped from TSA plates into Chelex-100-treated RPMI (C-RPMI) and normalized to an OD at 600 nm (OD_600_) of 1.0. Cells were washed in C-RPMI by centrifugation at 4,000 ***g*** for 8 min. Cells were diluted 1:1,000 in C-RPMI, and a further 1:100 dilution was made into the following conditions: RPMI, C-RPMI, C-RPMI+ions and C-RPMI+ions+FeCl_3_ to a final volume of 200 µl. Plates were incubated for 17 h at 37°C with shaking (180 r.p.m.). The OD_600_ was measured using a TECAN Infinite 200 PRO plate reader microplate reader. Where necessary, urine from pooled human donors (Medix Biochemica, CAT NO: 991-03-P) was supplemented at 10% into conditions.

### Whole human blood survival assay

Whole human blood was collected into heparin-coated vacutainers to prevent coagulation. The bacteria were grown overnight in TSB, washed twice in PBS and resuspended at 10^6^ c.f.u. ml^−1^. A final inoculum of 10^5^ c.f.u. ml^−1^ was obtained by inoculating 90 µl of whole human blood with 10 µl bacteria in a 96-well plate. Plates were incubated at 37°C with shaking (180 r.p.m.), and tenfold serial dilutions of samples in PBS were plated on TSA after 0, 0.5, 1, 2 and 4 h to determine c.f.u. The percentage survival was determined by comparing the number of c.f.u. at each time point to the initial number of c.f.u.

### Statistical analyses

Data were presented as the mean±sd. All experiments consisted of at least three independent biological repeats and were analysed by one-way ANOVA, two-way ANOVA, Mann–Whitney test of comparison, Kruskal–Wallis test or t-test (GraphPad Prism v10.5.0) as described in the figure legends. Genome-wide association analysis was performed using linear regression to assess associations between genetic variants (SNPs) and bacterial growth under iron-limited conditions. Analyses were conducted both with and without adjustment for population structure. To account for population stratification, the first two principal components derived from principal component analysis of the genotype data were included as covariates in the regression model. We only considered SNPs with a minor allele frequency of >5%. Statistical significance was determined at an uncorrected significance threshold of 0.05 given that lead variants were subsequently subjected to experimental functional validation.

## Results and discussion

### Establishment of a low-iron environment for *S. aureus* growth

**Fig. 1.**
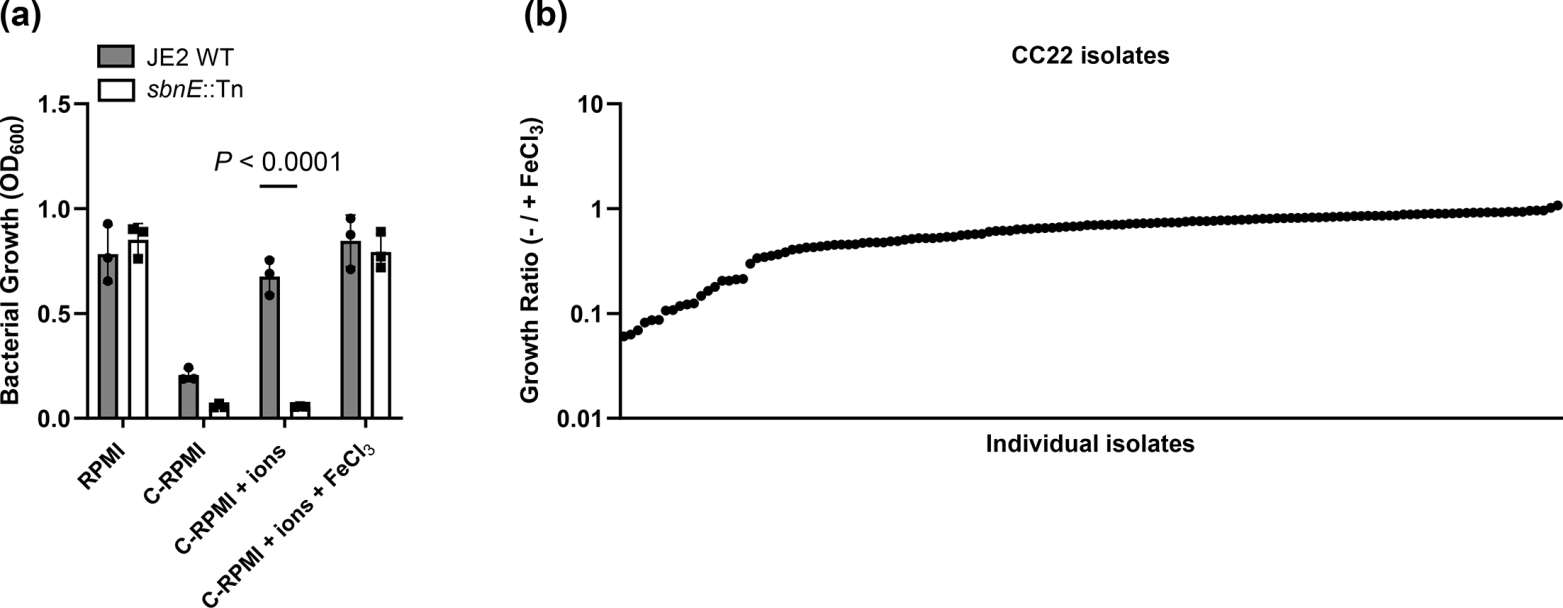
Significant variation exists across a collection of clinical SAB isolates in their ability to grow in a low-iron environment. (**a**) The ability of a wild-type (JE2 WT) and an isogenic siderophore mutant (JE2 *sbnE::*Tn) to grow in four growth media with varying levels of iron was compared. Their growth was equivalent and not statistically significantly different across three of the media, but in C-RPMI+ions which is replete with cations with the exception of iron, there was a significant difference in growth between the two strains. (**b**) The ability of a collection of 134 clinical bacteraemia isolates to grow with and without iron was quantified and the mean ratio for each presented. The OD_600_ was measured after 17 h and represents the final culture density. Data in (a) represents the mean±sd of three independent replicates. Data in (b) represents the mean of three independent replicates for each isolate. Data in (a) was analysed by a two-way ANOVA with Sidak’s post hoc test for multiple corrections.

To determine the importance of the ability to grow in a low-iron environment, we utilized a wild-type *S. aureus* strain (JE2 WT) and an isogenic mutant where *sbnE*, a gene required for synthesis of the staphyloferrin B siderophore, was inactivated by transposon insertion (JE2 *sbnE*::Tn) as our control strains [15]. We then developed four growth media: a base media of RPMI 1640 with 1% casamino acids (RPMI), RPMI with the ions chelated out (C-RPMI), C-RPMI where the ions other than iron were reintroduced (CRPMI+ions) and CRPMI+ions where iron was also reintroduced (C-RPMI+ions+FeCl_3_). The bacteria were grown in these media at 37°C with shaking for 17 h, and their growth was determined by OD (OD_600_). Both the wild-type and siderophore mutant (JE2 *sbnE*::Tn) grew equivalently in RPMI, and as expected due to the lack of essential ions, neither strain grew well in C-RPMI (Fig. 1a). The restoration of the ions (in the absence of iron) restored the growth of the wild-type but not the *sbnE*::Tn mutant, while restoration of FeCl_3_ restored the growth of both strains. These growth dynamics demonstrated that CRPMI+ions is a good environment to interrogate the genetic basis of growth in low-iron conditions.

### Significant variability exists across a collection of SAB isolates in the ability to grow in a low-iron environment

To examine how variable the ability to grow in a low-iron environment is across a collection of clinical isolates, we utilized a collection of 134 bacteraemia isolates all belonging to the CC22 lineage [12]. Growth of each isolate was measured in triplicate in iron-deficient (C-RPMI+ions) and iron-replete (C-RPMI+ions+FeCl_3_) conditions, and the ratio of growth between conditions was calculated. We used this ratio as opposed to growth in the low-iron environment alone to rule out any iron-independent effects there might be to growth in C-RPMI+ions alone across our clinical isolates. There was a wide range of growth abilities across the collection, with a greater than tenfold difference in growth between the best and worst growers (Fig. 1b). As we had the genome sequence for each of these isolates, we performed a genome wide association study (GWAS) to identify genetic loci that were associated with these growth phenotypes. While this statistical approach identified several loci as significantly associated (Table 2), our validation approach of mutating the associated gene did not support a role for any of the GWAS-identified loci as contributing to or affecting the ability of the bacteria to grow in a low-iron environment compared to the growth ratio of 0.80 for the JE2 wild-type strain (one-way ANOVA with Dunnet’s post hoc test for multiple comparisons). As *proS* is an essential gene, the NTML lacks a corresponding transposon insertion mutant [16]. Therefore, no growth ratio is reported for this CC22 locus tag in Table 2.

**Table 2.** GWAS-identified loci that were associated with growth in the low-iron environment CC22 locus tag: refers to the gene label as defined in the reference genome for this clone: HO 5096 0412. Description: provides information on any previously described gene names or putative activity. -Log10(P): minus the base 10 logarithm of the uncorrected GWAS-derived *P* value. NTML: is the name assigned to the specific transposon insertion mutant in the NTML. Growth ratio (-/+ FeCl_3_): was the growth of each specific mutant in the low-iron environment divided by growth in the high iron environment. Not applicable (n/a).

CC22 locus tag	Description	-Log_10_(*P*)	NTML no.	Growth ratio (-/+ FeCl_3_)
SAEMRSA15_20540	*czrB* (cation transporter)	2.37	NE199	0.77
SAEMRSA15_01210	*cap5G* (capsular polysaccharide biosynthesis protein)	2.32	NE580	0.65
SAEMRSA15_19830	Hypothetical protein	2.32	NE1520	0.79
SAEMRSA15_19400	*sdrH* (serine-aspartate repeat-containing protein)	2.25	NE1840	0.82
SAEMRSA15_04310	Tetrapyrrole methylase family protein	2.23	NE867	0.78
SAEMRSA15_02120	Putative teichoic acid biosynthesis protein B	2.23	NE362	0.78
SAEMRSA15_03860	*metB* (cystathionine gamma-synthase)	2.23	NE60	0.79
SAEMRSA15_25540	Preprotein translocase subunit SecA	2.23	NE66	0.80
SAEMRSA15_04600	Hypothetical protein	2.23	NE1799	0.86
SAEMRSA15_24960	TetR family transcriptional regulator	2.15	NE1216	0.79
SAEMRSA15_20480	Hypothetical protein	2.08	NE31	0.62
SAEMRSA15_19490	*scrB* (sucrose-6-phosphate hydrolase)	2.08	NE469	0.71
SAEMRSA15_18100	CamS sex pheromone cAM373	2.08	NE1087	0.75
SAEMRSA15_03840	Sodium-dependent transporter	2.08	NE231	0.77
SAEMRSA15_05850	DNA-binding response regulator	2.08	NE481	0.80
SAEMRSA15_26080	*gidB* (glucose-inhibited division protein B)	2.08	NE141	0.83
SAEMRSA15_16750	*ribBA* (riboflavin biosynthesis protein)	2.08	NE573	0.85
SAEMRSA15_26000	ABC transporter ATP-binding protein	1.97	NE1768	0.62
SAEMRSA15_02900	MATE efflux family protein	1.97	NE405	0.71
SAEMRSA15_24700	Acyltransferase	1.97	NE72	0.71
SAEMRSA15_23400	Putative membrane protein	1.97	NE154	0.77
SAEMRSA15_10960	*proS* (prolyl-tRNA synthetase)	1.97	n/a	n/a
SAEMRSA15_06610	Putative iron compound ABC transporter, ATP-binding protein	1.97	NE269	0.85
SAEMRSA15_10360	*carB* (carbamoyl phosphate synthase large subunit)	1.61	NE1454	0.73
SAEMRSA15_17760	Hypothetical protein	1.60	NE1202	0.68
SAEMRSA15_15240	*accC* (acetyl-CoA carboxylase, biotin carboxylase)	1.50	NE1519	0.80
SAEMRSA15_06310	*saeS* (sensor histidine kinase SaeS)	1.38	NE1296	0.95
SAEMRSA15_22540	*tcaA* (teicoplanin resistance-associated membrane protein TcaA protein)	1.34	NE285	0.73

### Bacteraemia isolates that originated from a urogenital site showed reduced ability to grow in low-iron conditions

**Fig. 2.**
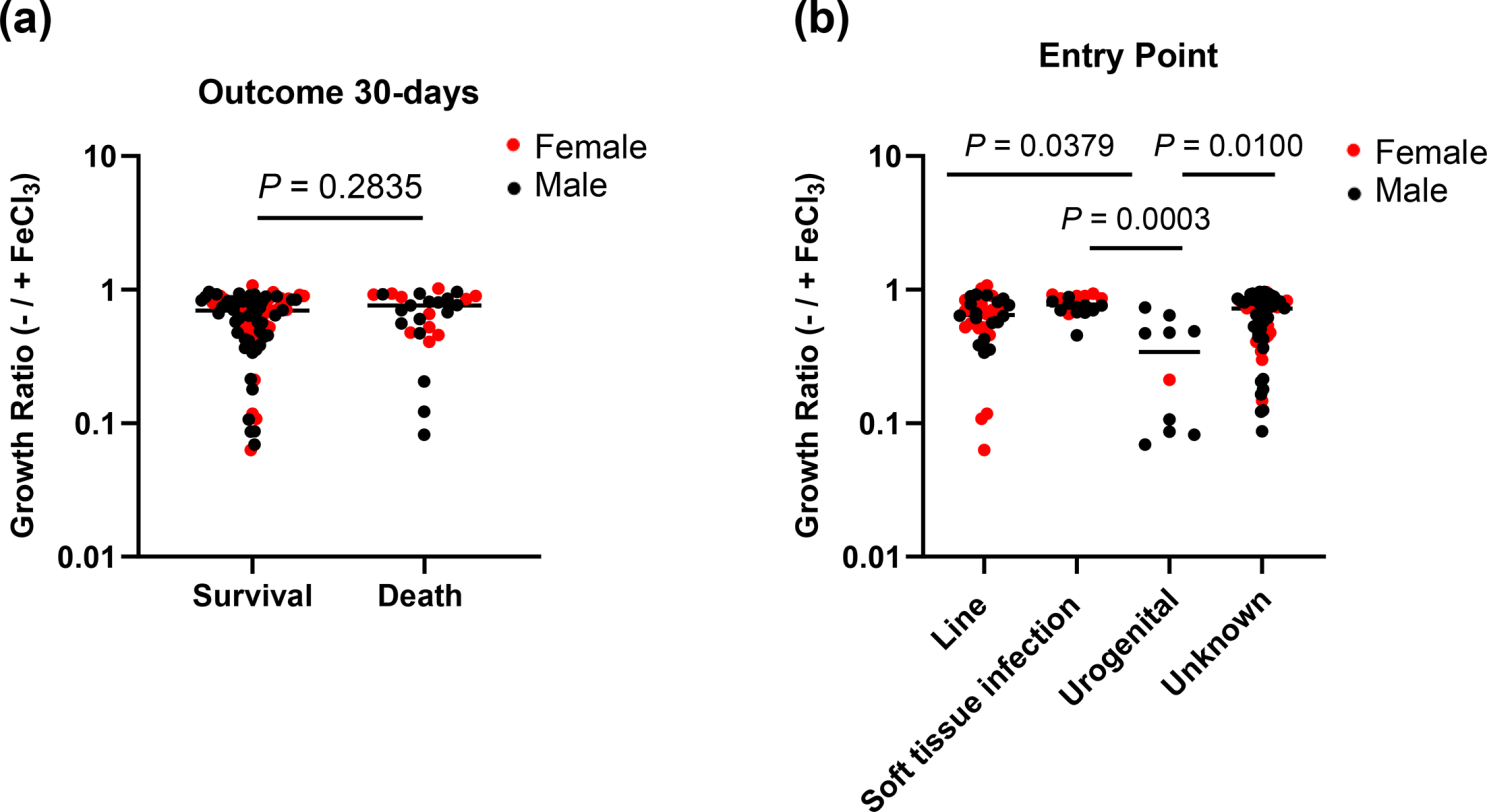
The inability to grow in a low-iron environment is associated with bacteraemia isolates that originated from a urogenital entry point. (**a**) The relative ability of the bacteria to grow in a low-iron environment was not associated with the 30-day mortality of the bacteraemic patients. (**b**) The proportion of bacteria that were unable to grow in a low-iron environment was significantly higher amongst isolates where the entry point into bloodstream was urogenital in origin, compared with those from an intravenous line, or a skin and soft tissue infection. Data in (a) and (b) represents the mean of three independent replicates for each isolate and the lines represent the means of all the strains. Data in (a) was analysed by Mann–Whitney test. Data in (b) was analysed by Kruskal–Wallis test with Dunnett’s post hoc test for multiple comparisons.

Some clinical data was available for this isolate collection, including the patient sex, 30-day survival and suspected entry point of the bacteria into the bloodstream [12]. There was no association between growth in a low-iron environment and patient sex (*P*=0.36) or the 30-day mortality rates (*P*=0.28) (Fig. 2a). There was however a significant association between a strain showing poor growth in low-iron conditions and whether the bacteraemia was urogenital in origin, with 50% (5/10) of the urogenital entry isolates showing poor growth with ratios of 0.4 and below (Fig. 2b), compared to less than 11% of strains with other entry points.

### Urine contains sufficient iron to facilitate the growth of all isolates

**Fig. 3.**
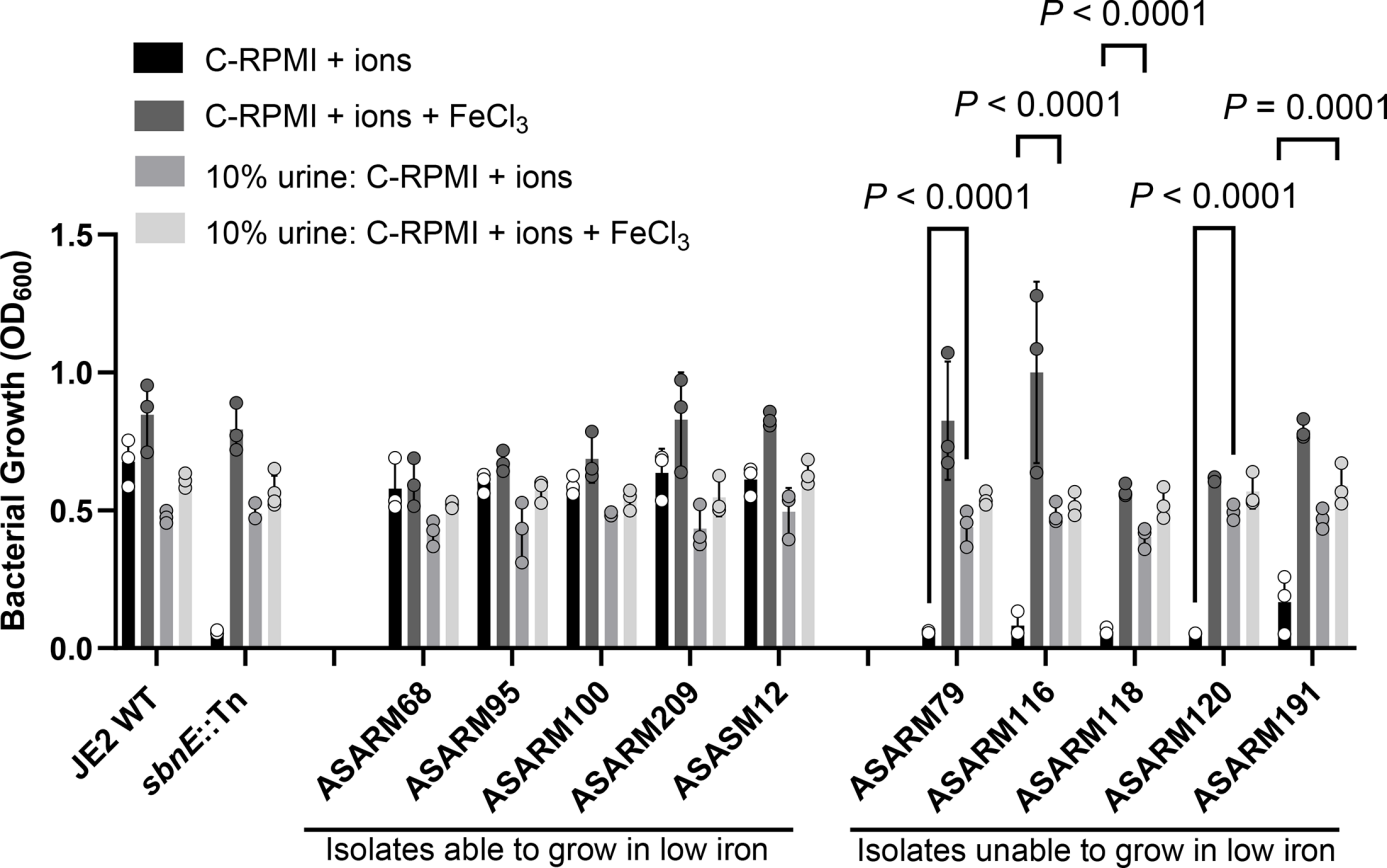
Urine supplementation facilitates the growth of the isolates otherwise unable to grow in a low-iron environment. The growth of the JE2 wild-type and *sbnE* mutant, alongside the ten urogenital origin isolates in C-RPMI+ions, C-RPMI+ions+FeCl_3_, C-RPMI+ions+10% urine and C-RPMI+ions+10% urine+FeCl_3_ was compared. Data represents the mean±sd of three independent repeats. Data was analysed by a two-way ANOVA with Tukey’s post hoc test for multiple comparisons.

The level of total iron in urine is relatively low when compared to blood. In healthy humans, urinary iron levels typically range from undetectable to 2.5 µM, whereas blood iron concentrations range from 7,500 to 10,000 µM [13,17,20]. By comparison, our iron-supplemented media contained 25 µM FeCl_3_, which was tenfold higher than that expected to be found in urine. Although relatively low, given the association of poor growth in low iron and a urogenital entry point into the bloodstream, we hypothesized that there may be sufficient iron in a form accessible to *S. aureus* in urine to enable these iron-dependent isolates to grow. To test this, we grew the wild-type and siderophore mutant alongside the ten urogenital origin isolates in C-RPMI+ions, C-RPMI+ions+FeCl_3_, C-RPMI+ions+10% urine, and C-RPMI+ions+10% urine+FeCl_3_. As anticipated, the wild-type and the five clinical isolates that were able to grow in the low-iron environment were able to grow in all these media (Fig. 3). However, the siderophore mutant and the five clinical isolates unable to grow in a low-iron environment continued to be unable to grow in the media C-RPMI+ions, but growth was restored by the addition of either FeCl_3_ or 10% urine, or both, suggesting that the level of iron present in urine is sufficient to complement the iron-associated growth defects of these isolates.

### The isolates with poor growth in the low-iron environment are also defective in their ability to survive in blood

**Fig. 4.**
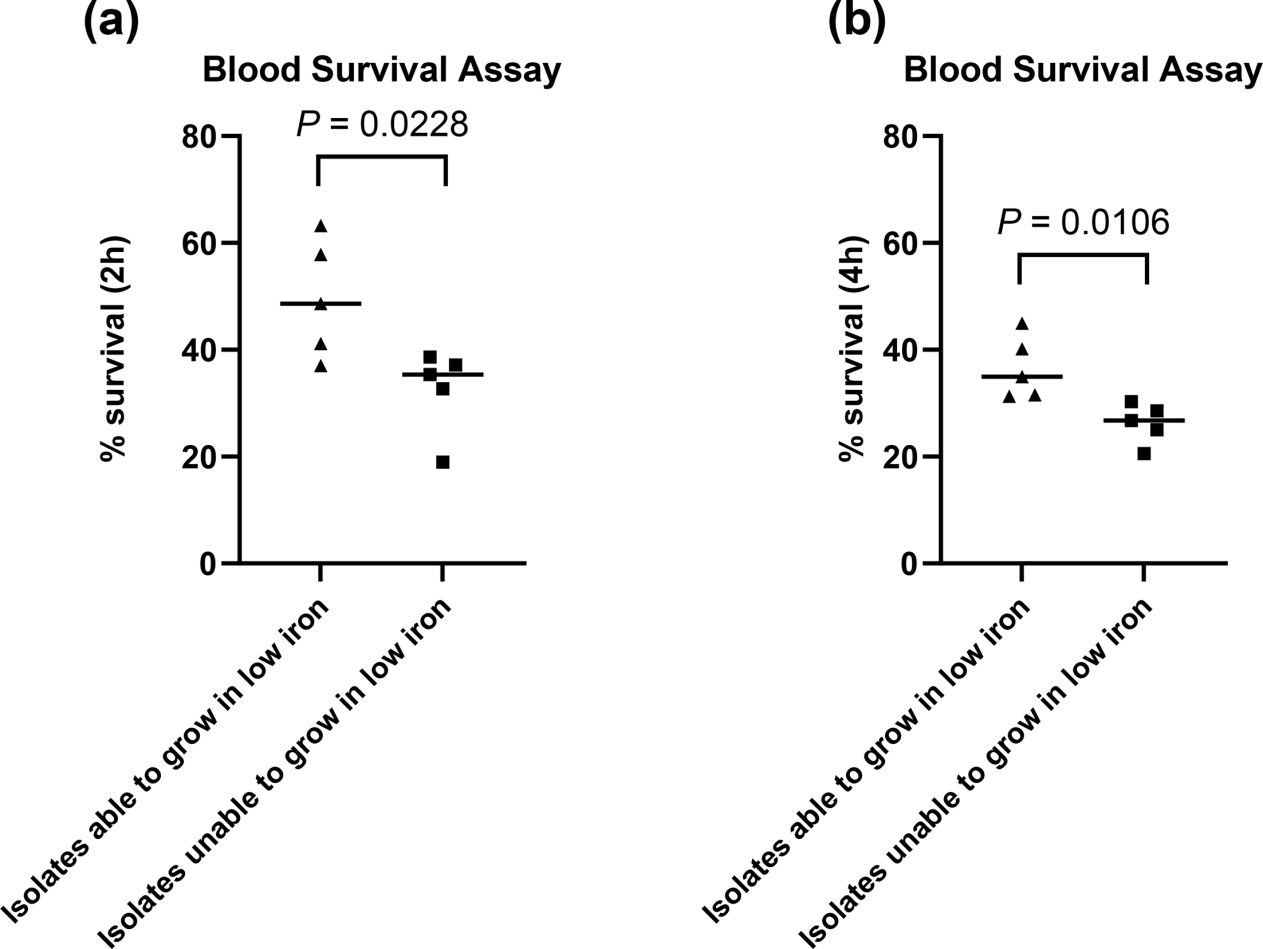
Clinical isolates unable to grow in low iron are also unable to survive in blood. The ten clinical bacteraemia isolates that were urogenital in origin were all exposed to fresh human blood for up to 4 h and their survival determined by plating out for c.f.u. ml^-1^. The five isolates able to grow in the low-iron environment were better able to survive in blood compared to the five that were unable to grow in the low-iron environment at 2 (**a**) and 4 (**b**) hours. Data represents the mean survival of at least three independent replicates for each isolate, and the lines represent the median of all the strains. Data was analysed by an unpaired, two-tailed t-test on the five biological replicates.

Nutritional immunity with respect to limiting the access of pathogens to nutrients such as iron is well established, especially in blood, where it is sequestered within molecules such as transferrin and haem, and very little free iron circulates around the bloodstream [9,21]. As such, bacteria must be able to scavenge iron to survive and grow in this environment. It was therefore surprising that isolates unable to grow in a low-iron environment were able to cause bacteraemia and that there was no association between the ability to grow in a low-iron environment and patient mortality. Blood represents a harsh environment for any bacteria to adapt to, and we hypothesized that despite their iron limitations, these isolates must be sufficiently robust to survive this exposure. To test this, we quantified the ability of the ten urogenital entry point bacteraemia isolates to survive in human blood for up to 4 h (Fig. S1Supplementary Material 1, available in the online Supplementary Material). We found that the survival of the isolates unable to grow in a low-iron environment was significantly lower than that of the isolates that were able to grow in the low-iron environment at 2 and 4 h (Figs 4 and S2).Supplementary Material 1 This suggests that they are poorly adapted to this environment, although at this point, whether this is directly due to their inability to grow in a low-iron environment or due to other factors such as increased susceptibility to the antibacterial factors found in blood is unclear.

## Conclusions

One of the many routes that *S. aureus* takes to enter the bloodstream and cause bacteraemia is through urogenital catheters [1,22]. Our findings here suggest that the urinogenital environment selects for variant isolates that have become dependent upon the exogenous provision of iron and also appear to be defective at surviving the initial exposure to human blood. Compared to blood, the levels of iron in urine are relatively low, and recent transcriptomic work supports the need of the bacteria to rapidly adapt to this iron-poor environment, where there was found to be a rapid upregulation of iron transporter systems by *S. aureus* following exposure to human urine [23]. It is therefore surprising that variants that are dependent upon exogenous sources of iron arise here, and even more surprising that despite being poorly able to survive exposure to blood, they were still able to establish themselves in the bloodstream and cause a bacteraemia. The underlying explanation for this apparent contradiction may lie in the underlying health problems of the infected host where both the need for catheterization and susceptibility to bacteraemia were present. It could alternatively be that some other benefit to the bacteria associated with iron dependency that is not currently apparent is at play. It was unfortunate that our GWAS and mutational validation approach did not identify any of the mutations that were responsible for this iron dependency, a likely effect of the low number of isolates with the defect within the isolate collection, and the polygenic nature of this phenotype, as this may have shone some light on this. However, what is clear is that the ability of *S. aureus* to cause bacteraemia is complex and multifaceted, and as there does not seem to be a ‘one-size fits all’ rule for how bacteraemia develops, it is likely that we are going to need tailored approaches to treatment based on both patient and pathogen features if we are to improve our ability to control these infections.

## Supplementary material

10.1099/mic.0.001691 Supplementary Material 1.

## Abbreviations

NTML: Nebraska Transposon Mutant Library
SAB: *Staphylococcus aureus* bacteraemia
TSA: tryptic soy agar
TSB: tryptic soy broth
