# Supplementary material for: Urogenital infections select for reduced ability to grow in a low-iron environment and reduced blood survival for Staphylococcus aureus
Source: Microbiology (Reading). 2026 Jun 8;172(6):001691. doi: 10.1099/mic.0.001691 (PMC13245813; doi:10.1099/mic.0.001691)
Supplement: Supplementary Material 1. [file mic-172-01691-s001.pdf]

Urogenital infections select for reduced ability to grow in a low iron environment and reduced blood survival for *Staphylococcus aureus*.

Kate P. Kearney[1,2], Elizabeth V. K. Ledger[1,2], Mario Recker[3,4] and Ruth C. Massey[1,2,5]*.

1: School of Microbiology, UCC, Cork, Ireland.

2: APC Microbiome Ireland, UCC, Cork, Ireland.

3: Centre for Ecology and Conservation, University of Exeter, Penryn Campus, Penryn, UK.

4: Institute for Tropical Medicine, University of Tübingen, Tübingen, Germany.

5: School of Cellular and Molecular Medicine, University of Bristol, Bristol, UK.

* for correspondence: r.massey@ucc.ie

Supplementary figures 1 and 2

Supplementary figure 1

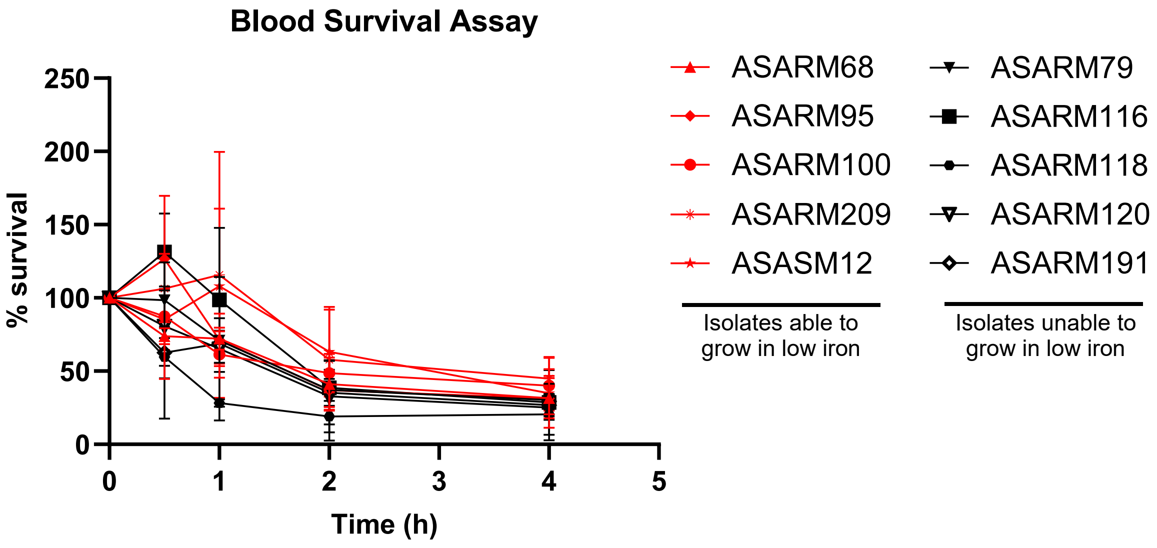

Supp. Fig. 1: The 10 urogenital entry point bacteraemia isolates were tested for their ability to survive in human blood for 0.5, 1, 2, and 4 hours. Isolates coloured in red correspond to isolates able to grow in low iron, while isolates coloured in black correspond to isolates unable to grow in low iron. The symbols represent the mean survival of at least two independent replicates for each isolate, and the error bars the standard deviation.

Supplementary figure 2

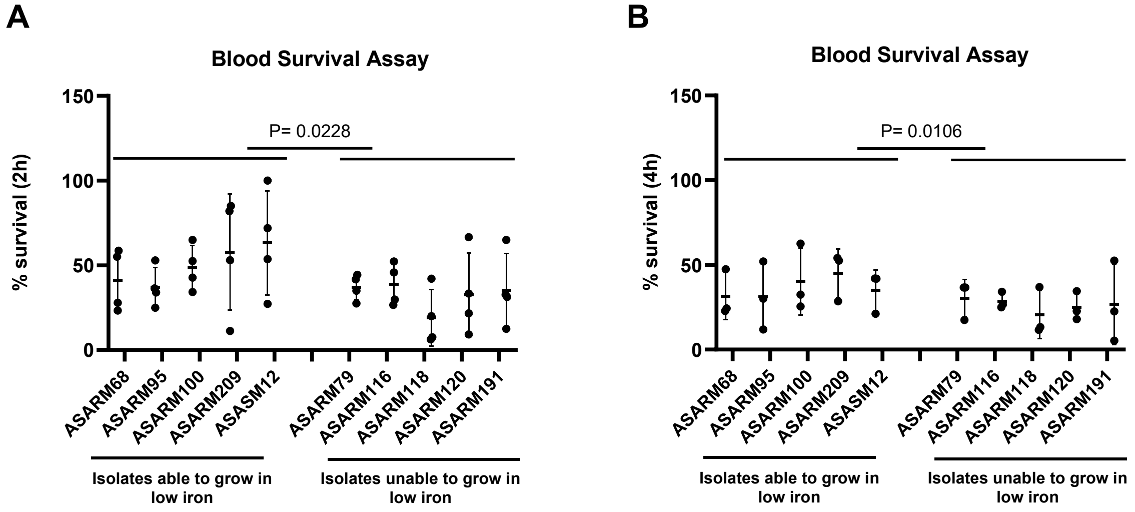

Supp. Fig. 2: Clinical isolates unable to grow in low iron are also unable to survive in blood. The ten clinical bacteraemia isolates that were urogenital in origin were all exposed to fresh human blood for up to 4 hours and their survival determined by plating out for CFU/ml. The five isolates able to grow in the low iron environment were better able to survive in blood compared to the five that were unable to grow in the low iron environment at 2 (A) and 4 (B) hours. The dots represent individual data points, the bars the mean value, and the error bars the standard deviation. Data was analysed by an unpaired, two-tailed t-test on the five biological replicates.
